# Mate Choice in *Mus musculus* Is Relative and Dependent on the Estrous State

**DOI:** 10.1371/journal.pone.0066064

**Published:** 2013-06-10

**Authors:** Léa Zinck, Susana Q. Lima

**Affiliations:** 1 Champalimaud Neuroscience Program, Champalimaud Centre for the Unknown, Lisbon, Portugal; 2 Instituto Gulbenkian de Ciência, Oeiras, Portugal; Tulane University Medical School, United States of America

## Abstract

Mate choice is a critical behavioral decision process with profound impact on evolution. However, the mechanistic basis of mate choice is poorly understood. In this study we focused on assortative mate choice, which is known to contribute to the reproductive isolation of the two European subspecies of house mouse, *Mus musculus musculus* and *Mus musculus domesticus*. To understand the decision process, we developed both full mating and limited-contact paradigms and tested *musculus* females' preference for *musculus* versus *domesticus* males, mimicking the natural *musculus/domesticus* contact zone. As hypothesized, when allowed to mate we found that sexually receptive *musculus* females exhibited a robust preference to mate with *musculus* males. In contrast, when non-receptive, females did not exhibit a preference and rather alternated between males in response to male mount attempts. Moreover in a no-choice condition, females mated readily with males from both subspecies. Finally, when no physical contact was allowed, and therefore male's behavior could not influence female's behavior, female's preference for its own subspecies was maintained independently of the estrous state. Together, our results suggest that the assortative preference is relative and based on a comparison of the options available rather than on an absolute preference. The results of the limited-contact experiments highlight the interplay between female's internal state and the nature of the interaction with prospective mates in the full mating conditions. With these experiments we believe we established an assortative mate preference assay that is appropriate for the investigation of its underlying substrates.

## Introduction

Sex is the essential feature of reproduction in most of the animal kingdom. Numerous factors influence mating, including genetic barriers or geographical proximity, but for most species there is an element of behavioral choice that is fundamental [1].

The process of choosing mates can be divided into three stages [2]: (1) reception of signals broadcast by prospective mates; (2) evaluation of the signals by receptive mates; (3) decision to either mate or reject. Many of the signals emitted by mates and used by choosers to decide have been identified, including coloration in fish [3]; song patterns in insects [4], birds [5] and amphibians [6]; and chemical signals in rodents [7]. From the receiver's side, the sensory pathways that detect and process these signals have been described for several systems, particularly for rodents [8]–[10]. In contrast, much less is known about the mechanisms of mate choice beyond the periphery, including how multiple mate signals are evaluated or how chooser's prior experience and internal state influence this complex decision-making process.

Several non-random mate preference patterns have been described for mice in laboratory conditions [11]–[13], including assortative mate preference whereby individuals choose to mate with phenotypically similar individuals. Assortative choice has been observed between conspecifics, and in some cases between conspecifics and heterospecifics mates [14]. This type of assortative mating has been shown to contribute to the pre-mating reproductive isolation of sibling species in distinct taxa [15]–[18], including the two subspecies of European house mouse, *Mus musculus musculus* and *Mus musculus domesticus* [19]–[23]. These two subspecies arose from a common ancestor and diverged recently by allopatric speciation (i.e. speciation occurring between populations that are geographically separated) following different human migratory flows that reached Europe from the Asian continent. *M. m. musculus* is found in Eastern Europe and Northern Asia, while *M. m. domesticus* occurs in the Mediterranean region and Western Europe. As a consequence, these two subspecies established a secondary contact zone in Central Europe, spanning from Denmark to the Black Sea [24] and studies with wild caught individuals showed an asymmetric assortative mate preference (only exhibited by animals of the *M. m. musculus* subspecies) [25], [26]. Together with other pre- and post-zygotic mechanisms [27]–[29], the assortative selection is thought to contribute to the reduced gene flow between *musculus* and *domesticus* individuals [30]. *Musculus* females use signals present in the urine and saliva of male mice to perform subspecies discrimination [19], [31]. However, similar to other studies of mate choice, little is known about the mechanisms underlying the behavioral choice exhibited by *musculus* females beyond sensory signaling.

We aimed to establish assortative mate preference of *M. m. musculus* females as an assay suitable for the subsequent investigation of its underlying brain mechanisms by using a combination of existing inbred wild-derived strains of mice together with laboratory strains. Like classical inbred strains, wild derived mice are well adapted to laboratory conditions and show low inter-individual variability. In contrast to the laboratory mice, however, wild-derived mice exhibit far richer behavioral repertoires [32]. To our knowledge, there is only one study with inbred wild-derived mice where full contact and mating was allowed [19]. However, due to abnormal female behavior of the particular *musculus* strain used, sexual interaction did not occur. Hence, we first investigated the behavioral choices of *musculus* females with the particular set of strains chosen in conditions where mating occurs. Because of controversy in the field regarding the influence of the estrous cycle on preference [33], we also examined the influence of the reproductive state on females' behavior and choice. In order to determine if female preference is absolute or relative, we tested whether female behavior depends on the set of choices offered. Finally, we compared preferences in the mate choice assay with those in a limited contact behavioral paradigm where animals can only perform social investigation, in order to test the effect of male behavior on female preference.

Here we show that inbred *musculus* females in the receptive estrous state prefer to associate and mate with *musculus* males, corroborating the assortative social preferences that have been reported for wild animals. However, the assortative preference disappears if females are in the non-receptive phase of the estrous cycle; and when no choice is permitted females interact and mate equally with either subspecies. These results suggest that *musculus* females exhibit a flexible behavioral preference that is not absolute but instead arises from the comparison of males from the two subspecies and which is modulated by the internal state. Moreover, we show that when only nose-nose contact is allowed *musculus* females prefer *musculus* males independently of their estrous state. This result highlights the need to take into consideration the interplay between the chooser's internal state and the type of interaction with prospective mates when investigating the mechanisms underlying mating behavior sequence.

## Materials and Methods

### Animals

As *musculus* representatives, we took advantage of the availability of two lines of the *musculus* subspecies, PWD/PhJ and PWK/PhJ strains, derived from animals trapped in the Czech Republic in 1972 and later inbred through sister/brother crossing in the laboratory [34]. These strains were ordered from The Jackson Laboratories and maintained in our animal facility. As a *domesticus* representative we used the classical inbred strain C57BL/6J, whose genome is primarily of *domesticus* origin [35]. This way we assured that the chooser, the *musculus* female, was of wild origin. Subject and stimulus mice used in this study were weaned at 21 days, housed in same-sex groups of two to four animals in stand-alone cages (1284L, Techniplast, 365×207×140 mm). Animals had access to food and water ad libitum and were maintained on a 12∶12 light/dark cycle (lights on at 0700). All experiments were performed during the dark phase of the cycle, under red dim light and began at least 2 hours after light offset. The home cage was changed every fortnight/alternate week to reduce the stress level of the animals; individuals were handled accordingly to Hurst and West 2010. Sexually naive females were tested between 2 to 4 months of age to assess their preference for sexually experienced males of the same age. All females tested belonged to the *M. m. musculus* PWD/PhJ strain, and pairs of male stimuli consisted of a *M. m. musculus* PWK/PhJ male and an age-matched *M. m. domesticus* C57BL/6J male.

To enhance female estrous cycle [36] and ensure a normal olfactory development of all our subject animals, we exposed them to male chemosensory signals every week, from weaning. To do so, male soiled bedding was mixed with clean bedding on the day of female's cage change, and in the alternate week, a filter paper containing 10 µL of male urine was placed in their home cage. Soiled bedding and urine consisted of a mix of equal volume from PWD/PhJ, PWK/PhJ, and C57BL/6J males. Vaginal smears allowed us to determine the estrous state of the females on the basis of the proportion of the cell types present in the smear [37]. In this study, we used both sexually receptive females, identifiable by a typical proestrous/estrous vaginal smear (characterized by the total absence of leukocyte and a mixture of nucleated epithelial cells and anucleated cornified cells) and non-receptive females showing metestrous or diestrous vaginal smears (characterized by the presence of leukocytes).

Males were isolated (individually housed) in stand-alone cages (1145T, Techniplast, 369×156×132 mm) two weeks before the beginning of the behavioral experiments to control their social rank. During this isolation period, males were given two sexual experiences of 2 hours with a PWD/PhJ female in proestrous-estrous. Only males that showed consistent sexual behavior, with several mounts, intromissions and at least one ejaculation during the two sexual trainings were used as stimulus males. The behavioral tests were performed using pairs of stimulus males from the PWK/PhJ and C57BL/6J strain which were matched to have the same date of birth, date of isolation, and date of sexual trainings. To limit the number of animals used in this study, stimulus males were re-used once in the social-preference test experiments and at the end of all experiments, test females were kept to train sexually stimulus males used in the next sets of experiments. All experiments were approved by the Animal Care and Users Committee of the Instituto Gulbenkian de Ciência, and the Portuguese National Authority for Animal Health (Direcção Geral de Veterinária).

### Behavioral Tests

In both behavioral paradigms the chooser was always a *Mus musculus musculus* female from the strain PWD/PhJ (*musculus* female) and the male stimuli were *Mus musculus musculus* from the strain PWK/PhJ (*musculus* male) and *Mus musculus domesticus* from the strain C57BL/6J (*domesticus* male). We chose the PWD/PhJ strain as choosers because the PWK females are known to exhibit some behavioral impairment during adulthood (from The Jackson Laboratories, and observation in our laboratory). Additionally, the *musculus* male stimulus was always from the PWK/PhJ strain (different from the test female), to avoid possible confounding effects from inbreeding avoidance and/or familiarity. Table S1 shows the different experiments that were performed in this study and their corresponding figure.

#### Partner-preference test

The partner-preference test (PPT) was adapted from Winslow's protocol [38]. The behavioral apparatus consisted of three transparent acrylic boxes (200×150×150 mm) connected by acrylic tubing of 30 mm diameter and 50 mm long. The floor of the three boxes was covered with clean bedding. In the center box, a clean disposable enrichment home (Datesand) containing a food pellet was added to increase the significance of this neutral area and to reinforce this free-choice test. To prevent male-male interactions (and fights) without limiting female interaction with each of the stimulus male, males were tethered in their box as described in Winslow (2003). Collars and tethers were constructed using fishing materials including nylon-coated steel wire leader and ball-swivel fast-locking snaps to allow free movement of the stimulus animal. They were carefully adjusted to the males' neck under slight isoflurane anesthesia during which it was ensured that the fit was secure and did not interfere with breathing. Tethered stimulus animals were acclimatized to their testing box for 15 minutes before the test, which was initiated by introducing either a receptive or a non-receptive female in the center box. Female estrous was determined by performing a vaginal smear at least 3 hours before testing. The PPT lasted for one hour during which an observer was constantly making sure that the tethered animals were behaving normally. Control experiments with a single stimulus male were also performed in the same conditions.

Video recording was performed using a Sony camera (HDR HC7E) connected to a computer running Virtual Dub software to acquire images. Off-line analysis of the behavior was performed semi-automatically using CleverSys, Inc. Annostar annotation program (Reston, VA, USA). Behavioral events were scored for the total duration of the test and were either related to the female's location or to male's sexual behavior. Female related measurements included number of entries into the male's compartment and time spent in *center*, *left* or *right* male box. Entries are defined as events where the female came from the center box to the male's compartment. Entries were further divided into re-entries (situations where the female returns to the same male's compartment) and switches (when the female goes to the other male's compartment). The female's preference score was calculated as *(amount time spent musculus male)/(total amount of time spent with the two males)*. Male sexual behaviors during female's presence in their box included: 1. *Mount attempt*: male attempt to copulate with the female by climbing onto her head, side or back; 2. *Mount*: after grabbing the female with both forepaws, the male climbs onto her from behind and restrains the female; 3. *Ejaculation*: male intense ‘freezing’, collapse and gripping of the female while performing a mount with intromission (as revealed by pelvic thrusts) and always followed by male self-grooming and a period of disinterest towards the female [39], [40]. As we could not distinguish mounts with intromissions from the ones without intromission, a mount was scored in both cases. Female's rejection to males' sexual behavior, characterized by an upright defensive posture of the female with or without vocalization, after a male mount attempt, were also scored.

#### Social-preference test

Social-preference tests (SPT) were conducted in behavioral apparatus designed similarly to the one used in the PPT, except that plastic partitions divided the male's box that impeded full contact between the male and female, preventing mating. The partition (1 mm thickness) had four holes of 8 mm diameter each, at its center, 20 to 40 mm from the floor, to allow facial contact and exchange of both volatile and non-volatile scents between the animals. The holes and plastic material allowed the females to gnaw and to pull at the barrier to gain access to the male which reflects female attraction for the male, as well as the measure of the time spent in proximity with the male [12]. The entire apparatus was cleaned and disinfected using Virkon® after each habituation and test, air-dried and covered with clean bedding each time. The females were habituated to the behavioral apparatus, without males, for 15 minutes for three days to reduce the stress levels of the animals and to ensure that females visited both sides and did not show any side bias over the three days of habituation. During the habituation days, at the end of 15 minutes, once the female was in the center box, the connections to male boxes were blocked and the females stayed for 5 minutes in the center box, prior to returning to their home cage. On the test day, the experiment was initiated with the same protocol used during the habituation days; however, during the 5 minutes that the female was forced to remain in the center box, the males were placed in their respective areas. Males were randomly assigned to the left or right compartment, in a balanced manner. The connections to the males' boxes were then reopened and the females were free to explore the entire box again for 15 minutes. After the test was finished, we performed vaginal smears to determine the estrous state of the females.

All experiments were recorded in similar manner as to PPT and female's location was then semi-automatically analyzed using CleverSys software. Three areas of interest were first manually delineated on a background image (*center*, *left* and *right* male box). Subsequently, the amount of time spent by the female in each area, the latency to enter the males' compartments and the number of entries were automatically quantified by the same software. Female's preference score was calculated as: *(amount time spent area of interest of musculus male)/(total amount of time spent in area of interest of the two male)*.

### Statistical analysis

Time spent by females near each male, both in the PPT and SPT, followed a normal distribution (Shapiro-Wilk test) and so two-way ANOVA tests were used to investigate the effect of male genotype (PWK/PhJ or C57BL/6J) and estrous state (Estrous or Diestrous) on the data variability. Otherwise we used non-parametric methods that rely on the rank of the data with no particular assumption. For paired samples (time spent by females near each male and number of visits to each male) we used Wilcoxon signed-rank tests. For independent samples, we used Mann-Whitney tests. Significance was accepted at *P*<0.05 (two-tailed) for all tests. Statistical analyses were carried out using Addinsoft XLSTAT-Pro software.

## Results

### Musculus females perform assortative mate choice when receptive

**Figure 1:**
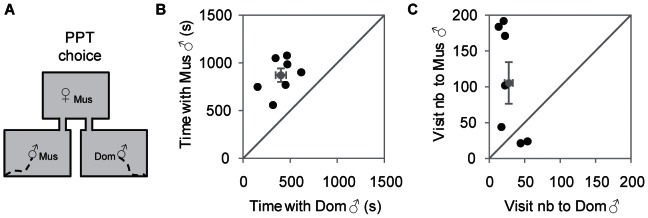
Sexually receptive *musculus* females prefer *musculus* to *domesticus* males in the Partner Preference Test. A) Schematic representation of the Partner Preference Test (PPT). B) Time spent in seconds by *musculus* females with each male (X ± SE *musculus* = 872±71 s; *domesticus* = 402±56 s; Wilcoxon test, *T* = 0, *N* = 7, *P* = 0.016). C) Visit number to each male by *musculus* females (X ± SE *musculus* = 105±29; *domesticus* = 27±6; Wilcoxon test, *T* = 24, *N* = 7, *P* = 0.109) (Black dots, female individual data; Grey dots, mean ± SE; Mus, *musculus*; Dom, *domesticus*).

**Figure 2:**
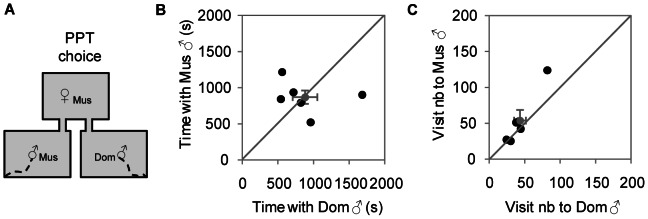
Sexually non-receptive *musculus* females do not prefer *musculus* to *domesticus* males in the Partner Preference Test. A) Schematic representation of the Partner Preference Test (PPT). B) Time spent in seconds by *musculus* females with each male (X ± SE *musculus* = 868±92 s; *domesticus* = 882±173 s; Wilcoxon test, *T* = 11, *N* = 6, *P* = 1). C) Visit number to each male by *musculus* females (X ± SE *musculus* = 54±15; *domesticus* = 43±8; Wilcoxon test, *T* = 4, *N* = 6, *P* = 0.208) (Black dots, female individual data; Grey dots, mean ± SE; Mus, *musculus*; Dom, *domesticus*).

During the 1-hour PPT all females (both in estrous and diestrous condition, Figure 1 and Figure 2) visited and contacted both available males. All *musculus* females in estrous exhibited a preference for *musculus* males that was detected in the time spent with each male (Figure 1b), the total number of entries to each male (Figure 1c) and preference score for *musculus* males (X ± SE = 0.69±0.03, N = 7). The number of re-entries into the *musculus* male area tended to be higher (X ± SE *musculus* = 89±29; *domesticus* = 11±6; Wilcoxon test, *T* = 24.00, *N* = 7, *P* = 0.108). Analysis of the females' behavior from the beginning of the session to the first mount (which took X ± SE = 774±130 seconds, *N* = 7) revealed that the assortative preference is already present at this stage (X ± SE preference score = 0.60±0.06, *N* = 7, Figure S1).

The assortative preference observed during the 1-hour PPT was mirrored at the level of sexual interactions, both in proportion of females that preferred to copulate with *musculus* males and the number of sexual attempts received from the *musculus* males (Table 1, Figure S2 for individual data). While all females received mounts from *musculus* males, only in two cases were the *domesticus* males successful in mounting the female; furthermore, only *musculus* males were able to reach ejaculation. In the case of the two females that received mounts from *domesticus* males, one occurred after the *musculus* male ejaculated, meaning that only one female chose to initiate sexual behavior with the *domesticus* individual (Figure S2).

**Table 1:** *Musculus* females perform assortative mate choice.

	Mean ± SE			N♀ (%)	
	Mus ♂	Dom ♂	*P*	Mus ♂	Dom ♂
Mount attempt	75±24	11±6	0.15	7 (100)	4 (57)
Female rejection	0±0	0±0	NS	0 (0)	0 (0)
Mount	7±1	3±2	0.18	7 (100)	2 (29)
Ejaculation	0.7±0.3	0±0	0.09	4* (57)	0 (0)

Male mount attempts, female rejection behavior, mounts, and ejaculations are presented in average (Mean ± SE) and for individual females (N_♀_ and percentage).

* indicates that one out of four females received 2 ejaculations from the *musculus* male during the one hour test. *P* values of Wilcoxon test comparing *musculus* and *domesticus* means are also presented (Mus, *musculus*; Dom, *domesticus*; NS, not significant).

### Musculus females do not exhibit assortative choice when they are non-receptive

Non-receptive *musculus* females did not exhibit an assortative preference, since they spent equal amount of time interacting with *musculus* and *domesticus* males (Figure 2b) and the number of visits to both males was the same (Figure 2c). During this time non-receptive females actively rejected the males' advances and no mounts were allowed in any of the experiments (Figure S2 and Table S2).

This is in contrast to what was observed for females in estrous. A two-way ANOVA, testing for main effects of male genotype and estrous condition on the duration of time spent by females with males showed a main effect of male genotype (Male effect: *F*_1,26_ = 5.82, *P* = 0.025), a main effect of the estrous condition (Estrous effect: *F*_1,26_ = 5.39, *P* = 0.030) and a significant interaction (Male*Estrous effect: *F*_1,26_ = 5.53, *P* = 0.028). This analysis is in agreement with the fact that the estrous state modulates the behavioral preference.

For the number of visits only the male genotype had a significant effect (Male effect: *F*_1,26_ = 6.86, p = 0.016; Estrous effect: *F*_1,26_ = 1.00, p = 0.328; and Male*Estrous effect: *F*_1,26_ = 3.57, p = 0.072). Since female rodents prefer to pace their interaction with males whenever possible, running away and returning to the male after each mount event [41], [42], we compared the entry behavior across reproductive states. Even though the total number of entries into the males' compartments was the same for females in the two reproductive states (X ± SE diestrous = 97±23; estrous = 133±25; Mann-Whitney test, *U* = 27, *N*_1_ = 6, *N*_2_ = 7, *P* = 0.445), the number of re-entries in both males' compartment tended to be higher in the estrous condition (X ± SE diestrous = 42±10; estrous = 100±26, Mann-Whitney test, *U* = 10.5, *N*_1_ = 6, *N*_2_ = 7, *P* = 0.145) while the number of switches was significantly higher in the diestrous condition (X ± SE diestrous = 26.7±3; estrous = 15.9±0.7; Mann-Whitney test, *U* = 10, *N*_1_ = 6, *N*_2_ = 7, *P* = 0.008). In other words, in the diestrous condition females alternated more between the males and after each switch they re-visited the male they had just interacted with less often.

### When no choice is allowed, musculus females mate equally with musculus and domesticus males

**Figure 3:**
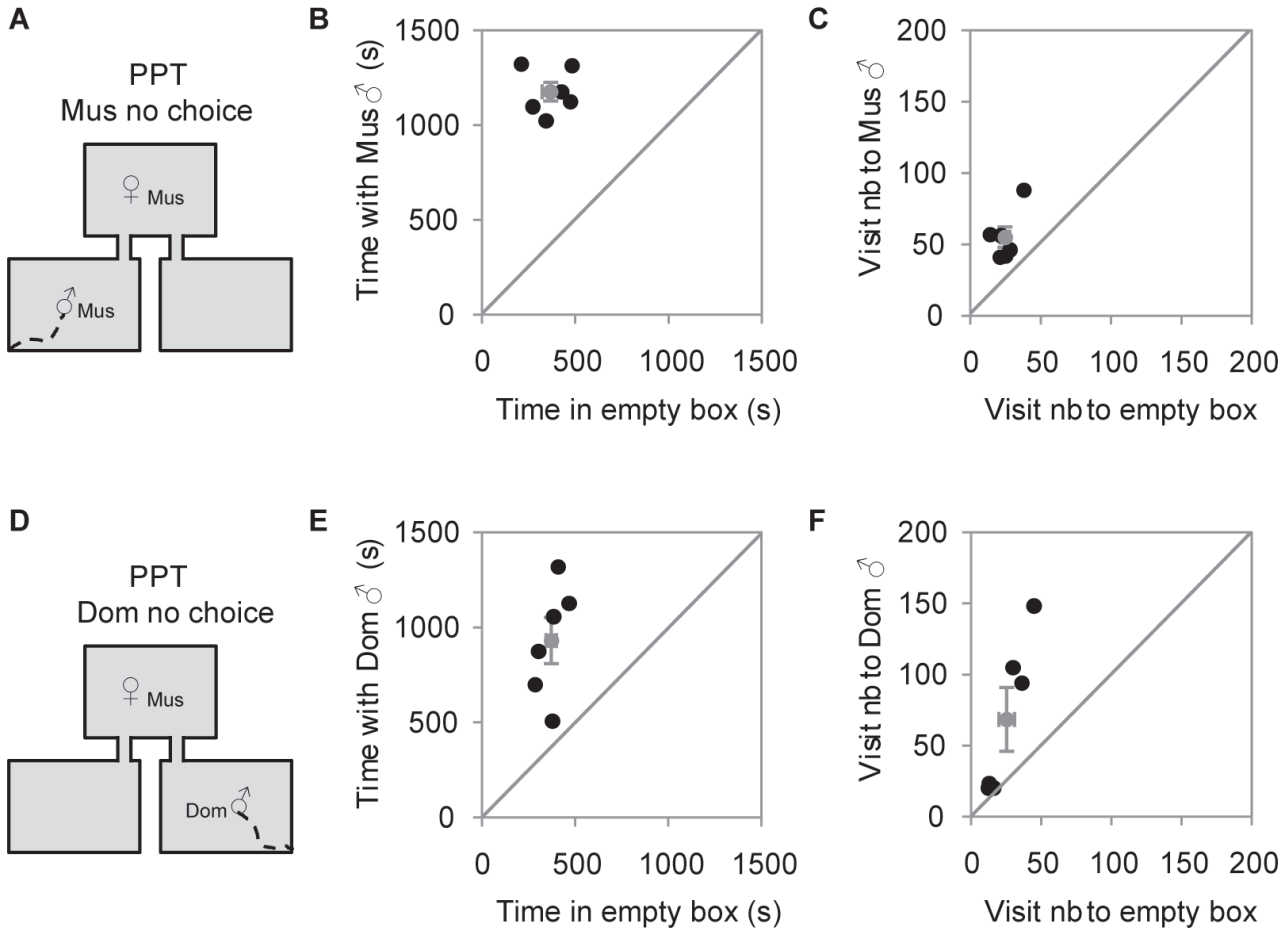
*Musculus* females are equally attracted by *musculus* **and** *domesticus* males in a no-choice condition. Schematic representation of the Partner Preference Test (PPT) with a *musculus* (A) or a *domesticus* male (D) and an empty box as alternative choice. B, E) Time spent in seconds by *musculus* females with a *musculus* or a *domesticus* male alone (X ± SE *musculus* = 1177±49 s; *domesticus* = 931±121 s; Mann-Whitney test, *U* = 9, *N*_1_ = 6, *N*_2_ = 6, *P* = 0.173). C, F) Visit number by *musculus* females to a *musculus* or a *domesticus* male alone (X ± SE *musculus* = 55±7; *domesticus* = 68±22; Mann-Whitney test, *U* = 18, *N*_1_ = 6, *N*_2_ = 6, *P* = 1) (Black dots, female individual data; Grey dots, mean ± SE; Mus, *musculus*; Dom, *domesticus*).

When only one male was present at the time (Figure 3a, 3d) females spent the same amount of time with each male (Figure 3b, 3e). The number of visits was also the same (Figure 3c, 3f). Analysis of behavior during the 1-hour test revealed that *musculus* females sexually interacted equally with both subspecies, as there were no rejection behaviors from the females, and no difference between the number of mount attempts, mounts or ejaculations received from each male in this no-choice scenario (Table 2).

**Table 2:** *Musculus* females mate equally with *musculus* and *domesticus* males in the no-choice condition.

	Mean ± SE			N_♀_ (%)	
	Mus ♂ alone	Dom ♂alone	*P*	Mus ♂alone	Dom ♂alone
Mount attempt	40.0±8.1	51.0±19.4	NS	6 (100)	6 (100)
Female rejection	0.0±0.0	0.0±0.0	NS	0 (0)	0 (0)
Mount	3.0±1.2	5.7±1.6	NS	5 (83)	6 (100)
Ejaculation	0.3±0.2	0.3±0.2	NS	2 (33)	2 (33)

Male mount attempts, female rejection behavior, mounts, and ejaculations are presented in average (Mean ± SE) and for individual females (N_♀_ and percentage). *P* values of Mann-Whitney test comparing *musculus* and *domesticus* means are also presented (Mus, *musculus*; Dom, *domesticus*; NS, not significant).

### Estrous independent assortative preference in a limited contact condition

**Figure 4:**
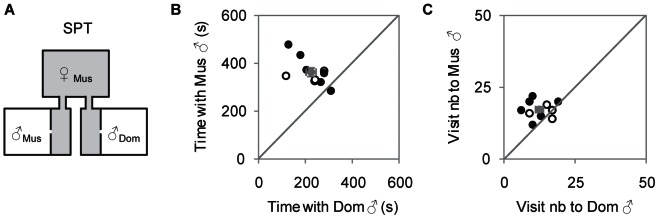
*Musculus* females prefer *musculus* to *domesticus* males in the Social Preference Test. A) Schematic representation of the Social Preference Test (SPT). B) Time spent in seconds by *musculus* females with each male (X ± SE *musculus* = 363±18 s; *domesticus* = 224±21 s; Wilcoxon test, *T* = 54, *N* = 10, *P* = 0.008). C) Visit number to each male by *musculus* females (X ± SE *musculus* = 17±1; *domesticus* = 13±1; Wilcoxon test, *T* = 41, *N* = 10, *P* = 0.033) (Black dots, diestrous females, individual data; Circles, estrous females; Grey dots, mean ± SE; Mus, *musculus*; Dom, *domesticus*).

Finally we investigated the behavior of receptive and non-receptive *musculus* females in a limited contact paradigm by performing Social Preference Tests (SPT, Figure 4a) that only allow nose-nose contact. Based on the average time it took for sexual behavior to start in the PPT (Figure S1), we performed SPT experiments for 900 seconds.

A two-way ANOVA, testing for main effects of male genotype and estrous condition on the duration of time spent by females with males showed a main effect of male genotype, but no significant effect of female estrous state and no significant interaction between these two factors (Male effect: *F*_1,20_ = 12.28, *P* = 0.003; Estrous effect: *F*_1,20_ = 0.33, *P* = 0.576; Male*Estrous effect: *F*_1,20_ = 0.24, *P* = 0.632). The same was observed for the number of visits as only the male genotype had a significant effect (Male effect: *F*_1,20_ = 14.51, *P* = 0.002; Estrous effect: *F*_1,20_ = 0.54, *P* = 0.473; and Male*Estrous effect: *F*_1,20_ = 3.57, *P* = 0.065). So, in contrast to what was observed during the PPT, the estrous state of the female had no influence on the male preference. The data for receptive and non-receptive *musculus* females during the SPT are shown together.

*Musculus* females exhibited a social preference for *musculus* males that was detected at the level of the time spent (Figure 4b), visit number to each male by *musculus* females (Figure 4c) and preference score for *musculus* males (X ± SE = 0.62±0.03, *N* = 10).

## Discussion

We established a behavioral paradigm suitable for investigating the mechanisms underlying mate choice. We took inspiration from the naturally occurring preference of *Mus musculus musculus* females for males of their own subspecies, which is thought to contribute to the reduced gene flow between the two European subspecies of house mouse, *Mus musculus musculus* and *Mus musculus domesticus*. Our strategy took advantage of existing wild-derived inbred strains of mice in combination with a regular laboratory inbred strain; and our testing included both full mating and limited-contact conditions. Full-mating tests, where females receive all signals that can affect their decision (in particular, somatosensory stimulation) are fundamental to unequivocally demonstrate female's choice and are rarely used in studies of mate choice [43]. These full-mating tests, where females chose between a *musculus* and *domesticus* male, showed a strong assortative choice. In contrast, if females were non-receptive, no preference was observed. If only one male was available at a time, females equally mated with both subspecies, suggesting that the choice relies on a flexible decision process. Finally, female preference during limited contact interaction was independent of the internal state.

We found a strong assortative preference of *musculus* females on estrous for *musculus* over *domesticus* males in the full mating condition: all females spent more time with the *musculus* male; 6 out of 7 females initiated their sexual interaction with a *musculus* male; and only *musculus* males reached ejaculation. In contrast, non-receptive females did not exhibit assortative preference. They vigorously rejected the males and/or ran away from the male box alternating to the other male after every male copulation attempt which led to increased time spent with both males. Furthermore, entries into the males' compartment were also qualitatively different across the estrous cycle. Females on estrous were more likely to re-enter the male compartment whereas diestrous females were more likely to switch male compartments. The decreased amount of time spent with males and the increased number of re-entries for estrous females might be related to the paced-mating behavior which was first observed for female rats [39] and which has also been documented for the mouse [42]. Paced mating, which happens when females can escape and control the amount of sexual stimulation they receive by interrupting males' mounts, is thought to reduce the aversive properties of mating; to be correlated with female arousal; and to be necessary for inducing a positive association with mating [41]. The finding that females perform more re-entries in estrous is indicative that they chose one male and kept escaping and re-visiting him, which decreased the total amount of time spent with males compared to the diestrous condition. Interestingly, diestrous females do not remain in their neutral box during the PPT. Mice value social contact [44] so diestrous females may still prefer to be next to a social stimulus, especially once the male stops trying to mount them. Further experiments with more naturalistic enclosures and different types of hideouts, from empty to potentially more rewarding stimuli (for example, female communal nest) will allow us to investigate if diestrous females have a lower overall preference for males relative to estrous females.

Receptive females in the “no choice” version of the PPT behaved similarly with males from either subspecies and were equally likely to achieve successful copulation. *Domesticus* males thus exhibit all the necessary features and behaviors for promoting *musculus* female arousal. This suggests that the *domesticus* male is only less valuable to the *musculus* female when it is actively compared to the *musculus* male and that the value of the two subspecies appears to be relative rather than absolute. This idea agrees with recent theoretical perspectives that mate choice should be considered in the context of other higher cognitive processes including learning and memory, inference and decision-making [2], [45], [46]. Despite the similar outcome at the population level, the behavior of the pair *domesticus-musculus* in the no-choice condition tended to be more variable when compared to the *musculus-musculus* pair. Further experiments are needed to understand if this higher variability is due to strain differences in male behavior or if they are an outcome of a difference in female motivation/arousal.

Insofar as mating could be dependent on specific “attractant” signals, males of both subspecies apparently have sufficient levels of these signals to reach a threshold to induce receptivity in the no-choice condition. However, males must emit different levels and females must be differentially sensitive to relative levels of these signals under conditions of comparison. Major urinary proteins (MUPs), which are molecules secreted in large quantities in male urine and are present in both *musculus* and *domesticus* subspecies, have been shown to be sufficient to underlie subspecies discrimination and recognition [7]. One recently identified MUP, darcin, a mouse pheromone capable of promoting attraction towards males carrying it, is a good candidate [47]. Another relevant chemical signal is the exocrine gland-secreting peptide 1 (ESP1), a mouse pheromone secreted in male tears [10] and which increases female receptivity. It is not known if PWK/PhJ males secrete more darcin/ESP1 compared to C57BL/6J males. If so, and if female sensitivity to these molecules is concentration dependent, darcin and ESP1 could help to explain the observed preference patterns. Further experiments will be needed to test these possibilities.

SPT experiments yielded indistinguishable results across the reproductive state, in marked contrast to the strong effect of estrous state observed during the PPT. Thus, the estrous state can have an influence on the behavioral choice of *musculus* females depending on the interaction allowed between individuals, a fact that has been inconsistent across previous studies using wild and wild-derived strains to test female preferences. While some studies have suggested that the estrous state has no influence on mouse female choice [19], [23], [31], others have suggested the opposite [33], and some have ignored the estrous state of the female entirely [26]. By comparing the behavior of females of the same strain in response to the same pair of males across the reproductive cycle, we found that the female's internal state influenced the female's behavior, not only affecting the dynamics of interaction with the male but also their overall preference. Whereas for diestrous females, males made several mount attempts leading to vigorous female rejection in the PPT, in SPT the males were prevented from mount attempts. We suggest that expression of assortative preference, regardless of reproductive state, as observed in SPT, may be the default. The absence of preference in diestrous females in the PPT could result from aversion triggered by the male “aggression” of mounting attempts. Because of the limited contact, the SPT offers the possibility of observing female's behavior in the absence of male's actions (although odors and vocalizations are still present) and can be regarded as a proxy for the appetitive phase where animals sample the signals present in the environment. This contrasts to the PPT, which models not only the appetitive phase, but also what happens during the consummatory period during which the males' behavior may influence female's choice.

We believe this study validates the use of wild-derived strains for a simple and reliable mate choice assay in both limited-contact (SPT) and full-interaction (PPT) paradigms. This combination of resources offers a promising avenue for unraveling some of the mechanisms underlying the biologically relevant behavioral process of assortative mate choice. Although most transgenic mouse lines such as Cre recombinase-lines, now widely used for tissue-specific manipulations [48], are only available in classical laboratory strains (C57B/6J, etc.), these can be backcrossed to the wild-derived background and, in principle, all available genetic resources can be used in these strains. Combining viral vectors with tissue-specific promoters may also allow efficient expression of exogenous proteins, permitting optogenetic and other manipulations of defined neuronal populations in wild-derived strains. By comparing SPT and PPT with the same strains of animals, we were able to tease apart the effect of the male's behavior on female choice and its interaction with the female's internal state. These paradigms will allow us to investigate the neural mechanisms underlying mate preferences in near-natural conditions, while at the same time maintaining an experimentally tractable assay.

## Supporting Information

Figure S1 ***Musculus*** **females exhibit a preference for** ***musculus*** **males both during the approach and the copulatory phases.** Assortative mate preference can be seen both before (A, B) and after (C, D) the first male mount occurred. Time spent by *musculus* females with each male, before (B, X ± SE, *musculus* = 214±31 s; *domesticus* = 131±16 s; Wilcoxon test, *T* = 23, *N* = 7, *P* = 0.151) and after (C, X ± SE, *musculus* = 654±87 s; *domesticus* = 271±54 s; Wilcoxon test, *T* = 28, *N* = 7, *P* = 0.022) the first male mount occurred. Visit number to each male by *musculus* females, before (B, X ± SE, *musculus* = 20±8; *domesticus* = 8±1; Wilcoxon test, *T* = 18, *N* = 7, *P* = 0.142) and after (D, X ± SE, *musculus* = 86±24 s; *domesticus* = 20±6; Wilcoxon test, *T* = 24, *N* = 7, *P* = 0.108) the first male mount occurred (Black dots, female individual data; Grey dots, mean ± SE; Mus, *musculus*; Dom, *domesticus*).
(TIF)

Figure S2 **Individual behavioral sequence of estrous and diestrous females during Partner Preference Tests.** Each row describes the behavioral events occurring, as a function of time during PPT, between a sexually receptive (A) or non-receptive (B) *musculus* female and a *musculus* or a *domesticus* male (open and closed symbols, respectively). The first visit to each male (square), the first male mount attempt (triangle), every male mount (diamond) and ejaculation (circle) as well as female switch from one male to the other (dot) are represented (Mus, *musculus*; Dom, *domesticus*).
(TIF)

Table S1 **Partner Preference Tests and Social Preference Tests performed in this study.** PPT, Partner Preference Test; SPT Social Preference Test; Mus, *musculus*; Dom, *domesticus*.
(DOC)

Table S2 ***Musculus*** **females do not exhibit male preference during the Partner Preference Test when they are not sexually receptive.** Male mount attempt, female rejection behavior. mount, and ejaculation are presented in average (mean ± SE) and for individual females (N_♀_ and percentage). *P* values of Wilcoxon test comparing *musculus* and *domesticus* means are also presented (Mus, *musculus*; Dom, *domesticus*; NS, not significant).
(DOC)
