# Supplementary figures and images for: Mate Choice in Mus musculus Is Relative and Dependent on the Estrous State
Source: PLoS One. 2013 Jun 10;8(6):e66064. doi: 10.1371/journal.pone.0066064 (PMC3677927; doi:10.1371/journal.pone.0066064)

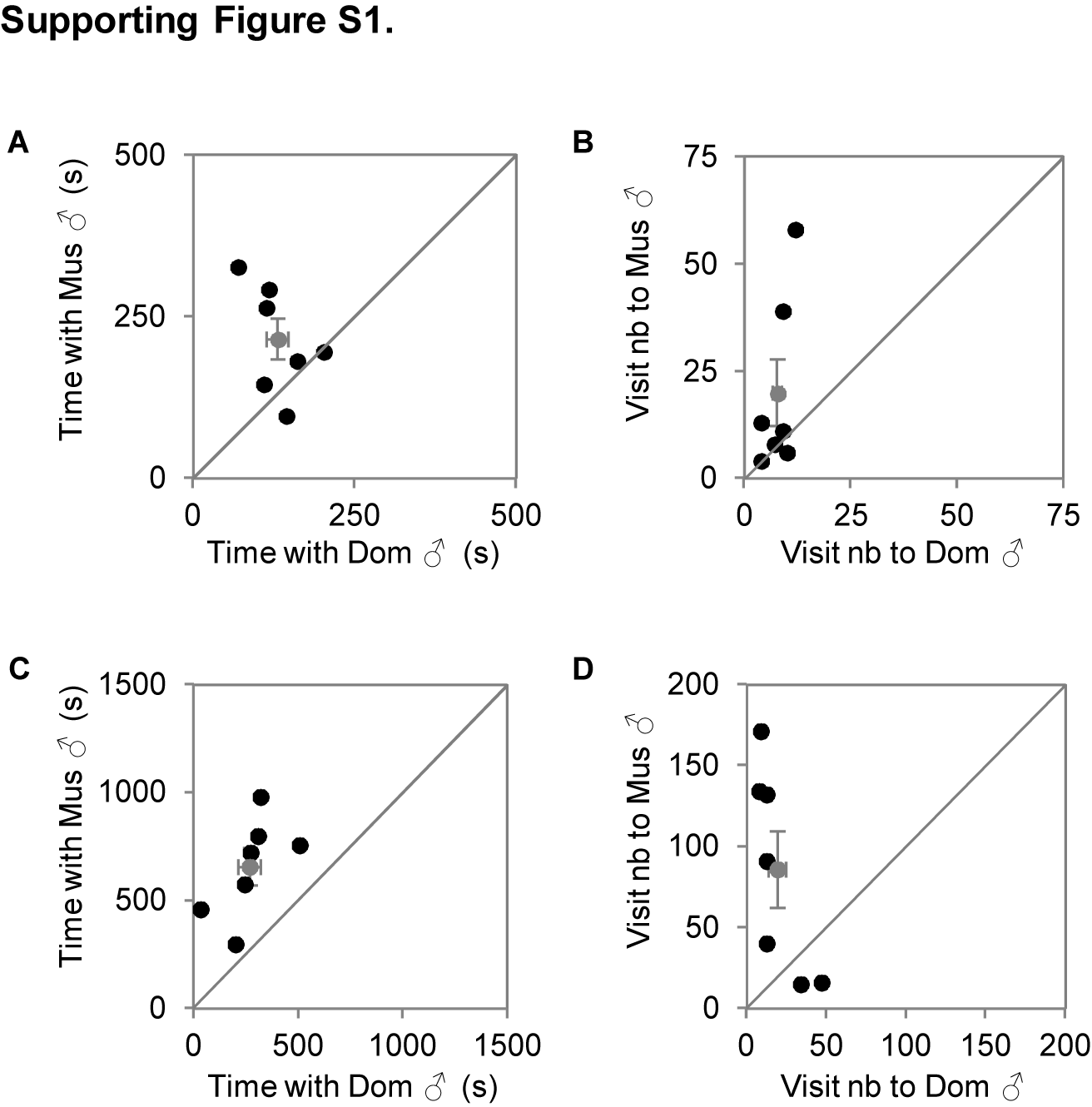

Supplement: Figure S1 — Musculus females exhibit a preference for musculus males both during the approach and the copulatory phases. Assortative mate preference can be seen both before (A, B) and after (C, D) the first male mount occurred. Time spent by musculus females with each male, before (B, X ± SE, musculus = 214±31 s; domesticus = 131±16 s; Wilcoxon test, T = 23, N = 7, P = 0.151) and after (C, X ± SE, musculus = 654±87 s; domesticus = 271±54 s; Wilcoxon test, T = 28, N = 7, P = 0.022) the first male mount occurred. Visit number to each male by musculus females, before (B, X ± SE, musculus = 20±8; domesticus = 8±1; Wilcoxon test, T = 18, N = 7, P = 0.142) and after (D, X ± SE, musculus = 86±24 s; domesticus = 20±6; Wilcoxon test, T = 24, N = 7, P = 0.108) the first male mount occurred (Black dots, female individual data; Grey dots, mean ± SE; Mus, musculus; Dom, domesticus). (TIF) [file pone.0066064.s001.tif]

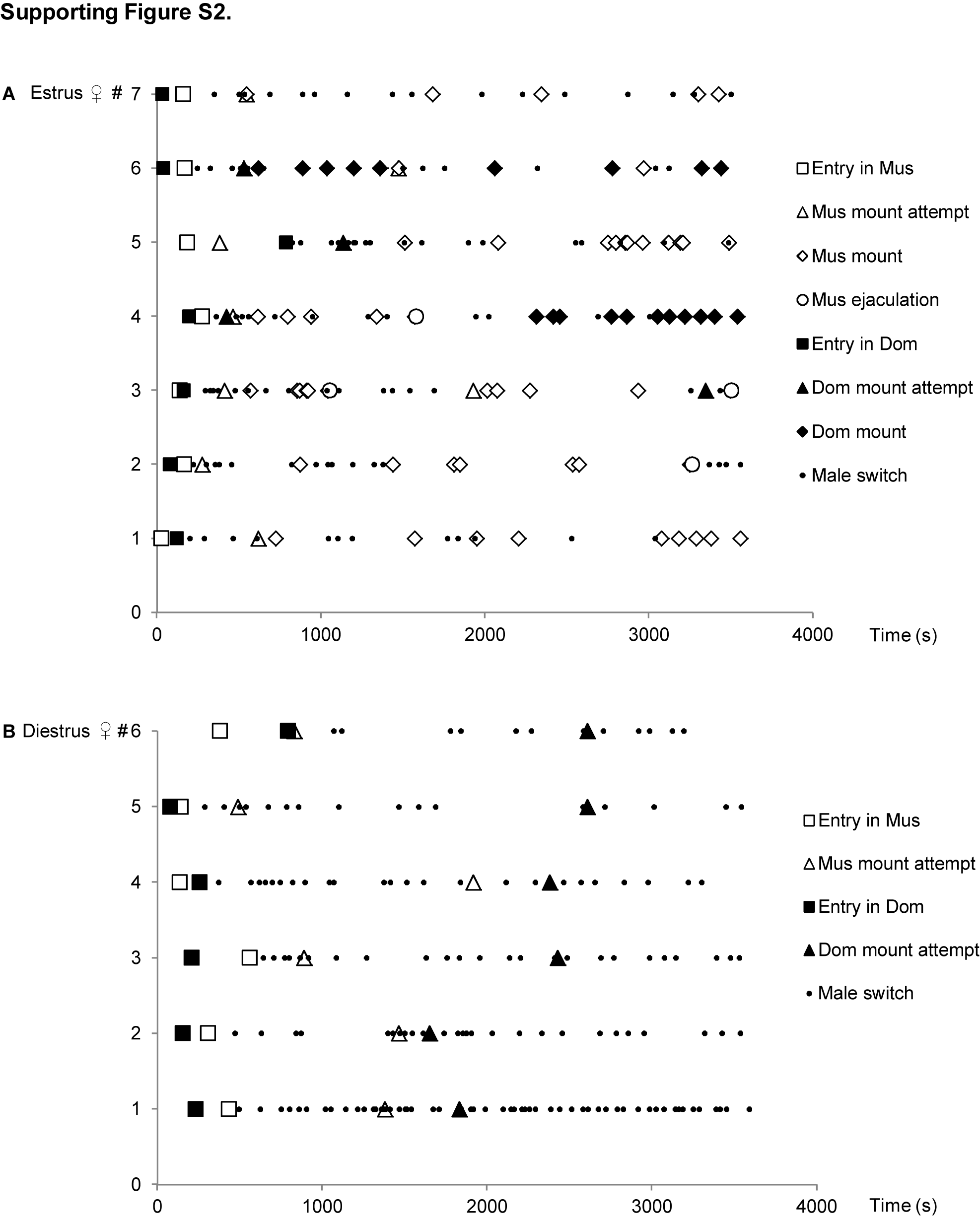

Supplement: Figure S2 — Individual behavioral sequence of estrous and diestrous females during Partner Preference Tests. Each row describes the behavioral events occurring, as a function of time during PPT, between a sexually receptive (A) or non-receptive (B) musculus female and a musculus or a domesticus male (open and closed symbols, respectively). The first visit to each male (square), the first male mount attempt (triangle), every male mount (diamond) and ejaculation (circle) as well as female switch from one male to the other (dot) are represented (Mus, musculus; Dom, domesticus). (TIF) [file pone.0066064.s002.tif]
