# Supplementary material for: Mate Choice in Mus musculus Is Relative and Dependent on the Estrous State
Source: PLoS One. 2013 Jun 10;8(6):e66064. doi: 10.1371/journal.pone.0066064 (PMC3677927; doi:10.1371/journal.pone.0066064)
Supplement: Table S1 — Partner Preference Tests and Social Preference Tests performed in this study. PPT, Partner Preference Test; SPT Social Preference Test; Mus, musculus; Dom, domesticus. (DOC) [file pone.0066064.s003.doc]

Supporting Table S1

|  | Fig 1 | Fig 2 | Fig 3A | Fig 3B | Fig 4 | |
| --- | --- | --- | --- | --- | --- | --- |
| Behavioral apparatus | PPT | PPT | PPT | PPT | SPT | |
| Stimulus ♂ | Mus vs Dom | Mus vs Dom | Mus alone | Dom alone | Mus vs Dom | |
| ♀ state | Receptive | Non-receptive | Receptive | Receptive | Receptive | Non-receptive |
