# Supplementary material for: Mate Choice in Mus musculus Is Relative and Dependent on the Estrous State
Source: PLoS One. 2013 Jun 10;8(6):e66064. doi: 10.1371/journal.pone.0066064 (PMC3677927; doi:10.1371/journal.pone.0066064)
Supplement: Table S2 — Musculus females do not exhibit male preference during the Partner Preference Test when they are not sexually receptive. Male mount attempt, female rejection behavior. mount, and ejaculation are presented in average (mean ± SE) and for individual females (N♀ and percentage). P values of Wilcoxon test comparing musculus and domesticus means are also presented (Mus, musculus; Dom, domesticus; NS, not significant). (DOC) [file pone.0066064.s004.doc]

**Supporting Table S2.**

|  | **Mean ± SE** | |  | **N♀ (%)** | |
| --- | --- | --- | --- | --- | --- |
|  | **Mus ♂** | **Dom ♂** | ***P*** | **Mus ♂** | **Dom ♂** |
| Mount attempt | 20 ± 7 | 6 ± 2 | NS | 6 (100) | 6 (100) |
| Female rejection | 19 ± 6 | 6 ± 1 | NS | 6 (100) | 6 (100) |
| Mount | 0 ± 0 | 0 ± 0 | NS | 0 (0) | 0 (0) |
| Ejaculation | 0 ± 0 | 0 ± 0 | NS | 0 (0) | 0 (0) |
